# Fibroblast growth factor inhibition by molecular-targeted agents mitigates immunosuppressive tissue microenvironment in hepatocellular carcinoma

**DOI:** 10.1007/s12072-023-10603-z

**Published:** 2023-10-21

**Authors:** Hiroyuki Suzuki, Hideki Iwamoto, Toshimitsu Tanaka, Takahiko Sakaue, Yasuko Imamura, Atsutaka Masuda, Toru Nakamura, Hironori Koga, Yujin Hoshida, Takumi Kawaguchi

**Affiliations:** 1 Division of Gastroenterology, Department of Medicine, Kurume University School of Medicine, 67 Asahi-machi, Kurume, 830-0011 Japan; 2 Liver Cancer Research Division, Research Center for Innovative Cancer Therapy, Kurume University, Kurume, 830-0011 Japan; 3 Iwamoto Internal Medicine Clinic, Kitakyushu, 802-0832 Japan; 4 Division of Digestive and Liver Diseases, Department of Internal Medicine, University of Texas Southwestern Medical Center, Dallas, TX 75390 USA

**Keywords:** Molecular targeting, Tumor immune microenvironment, Orthotopic, Fibroblast growth factor, Vascular endothelial growth factor receptor, Hepatocellular carcinoma, Immune checkpoint inhibitor, *APOC1*, *VIPR1*, RNA sequencing

## Abstract

**Background & aims:**

Combination immunotherapy refers to the use of immune checkpoint inhibitors (ICI) and molecular-targeted agents (MTA), which have recently been approved for the treatment of advanced hepatocellular carcinoma (HCC). Owing to its relatively low antitumor effect (up to 30%), sequential therapy following ICIs treatment is required in patients with HCC. This study aimed to determine the impact of MTAs on the tumor immune microenvironment (TIME).

**Methods:**

We established immune syngeneic orthotopic HCC mouse models using Hep-55.1C and Hep-53.4, and treated them with MTAs (lenvatinib, sorafenib, regorafenib, cabozantinib, and DC101 as anti-vascular endothelial growth factor receptor-2 antibodies, and AZD4547 as a fibroblast growth factor receptor (FGFR)-1/2/3/4 inhibitor) for 2 weeks. Subsequently, alterations in the TIME caused by MTAs were evaluated using immunohistochemistry (antibodies for CD3, CD8, Foxp3, Granzyme B, Arginase-1, NK1.1, F4/80, CD11c, PD-1, and PD-L1). We conducted RNA-seq analysis using lenvatinib- and AZD4547-treated tumors. To confirm the clinical relevance of these findings, we analyzed the transcriptome data of human HCC cells (MHCC-97H) treated with various concentrations of lenvatinib for 24 h using RNA-seq data from the Gene Expression Omnibus database.

**Results:**

The number of Foxp3- and F4/80-positive cells in the TIME was decreased in many MTAs. Cabozantinib increased the numbers in NK1.1-, Granzyme B, and CD11c-positive cells. Lenvatinib and AZD4547 increased the number of CD8, Granzyme B, and PD-L1-positive cells. Gene ontology enrichment analysis revealed that lipid metabolism-related genes were downregulated by lenvatinib and AZD4547. In total, 161 genes downregulated by FGFR inhibition in rodent models overlapped with those downregulated by lenvatinib in human HCC cells.

**Conclusions:**

In this study, we showed that cabozantinib activated the innate immune system, and lenvatinib and AZD4547, which commonly inhibit FGFR signaling, altered TIME to a hot immune state by downregulating lipid metabolism-related genes. These findings support the therapeutic use of combination immunotherapies.

**Supplementary Information:**

The online version contains supplementary material available at 10.1007/s12072-023-10603-z.

## Introduction

Hepatocellular carcinoma (HCC), the most common primary hepatic malignancy, is the fourth leading cause of cancer-related death worldwide [1]. Despite major therapeutic advances in recent decades, the 5-year survival rate for advanced HCC is poor at less than 10% [1]. Atezolizumab, an immune checkpoint inhibitor (ICI), along with bevacizumab, a molecular-targeted agent (MTA), tremelimumab (ICI), and durvalumab (ICI), have significantly influenced HCC treatment, establishing it as a front-line therapy for patients with advanced HCC. However, the antitumor effects of these treatments remain low (up to 30%) [2, 3]. The tumor immune microenvironment (TIME), a state of immunocompetent cells infiltrating the tumor microenvironment, correlates with the therapeutic efficacy of ICIs [4]. Therefore, regarding ICI therapy, the transformation of ‘immune cold tumors,’ in which immune cells hardly infiltrate, into ‘immune hot tumors,’ in which immune cells easily infiltrate, is believed to enhance the antitumor effects of ICIs [5]. HCC is an immune cold tumor that is resistant to ICIs, especially nonalcoholic steatohepatitis-related HCC [5, 6]. HCC is highly refractory to therapeutic interventions. Despite surgical resection or ablation, 70% of patients experience tumor recurrence within 5 years, and tumor control is difficult with monotherapy alone [7]. In real-world practice, implementing sequential therapy using various ICIs and MTAs has prolonged progression-free survival (PFS) and overall survival (OS); however, the appropriate sequential therapy following ICI therapy remains controversial [8, 9].

Combination immunotherapy, which combines ICI with local therapy (e.g., radiofrequency ablation and radiation therapy), systemic chemotherapy (e.g., MTA), and other therapies, has been used as a strategy to enhance the therapeutic effect of ICI in several types of cancer treatment, including HCC, and its high antitumor effect has been attracting attention [7]. MTAs are known to inhibit angiogenesis by inhibiting vascular endothelial growth factor (VEGF) signaling and directly or indirectly affecting macrophages, cytotoxic T cells, and regulatory T cells (Tregs), leading to dynamic changes in the TIME [5]. In addition to the common inhibition of VEGF signaling, MTAs have a unique inhibition profile (e.g., lenvatinib has relatively strong fibroblast growth factor (FGF) signaling inhibition and cabozantinib has particularly potent hepatocyte growth factor/c-Met signaling inhibition) [5]. Therefore, to generate a basic rationale for suitable MTAs for combination with ICIs, sequential ICIs, or combination immunotherapy, we aimed to evaluate the effect of MTA on TIME using an immune syngeneic orthotopic HCC mouse model.

## Materials and methods

### Experimental procedures

#### Cell lines and culture conditions

We purchased Hep-55.1C and Hep-53.4, mouse hepatoma cell lines, from Cell Line Service GmbH (Oppenheim, Germany). The human hepatoma cell line, HuH7 (JCRB0403) was purchased from the Japanese Collection of Research Bioresource (JCRB) Cell Bank (Tokyo, Japan). Hepatoma cells were maintained in Dulbecco’s modified Eagle’s medium (DMEM; Gibco, Invitrogen Cell Culture Co., Auckland, New Zealand) containing 10% fetal bovine serum (Biowest, Nuaille, France) and 100 units/mL penicillin–streptomycin (Nacalai Tesque, Inc., Kyoto, Japan).

#### Reverse transcription–quantitative real-time PCR

To evaluate the effect of MTAs on hepatoma cells (Hep-55.1C and HuH7), reverse transcription–quantitative (RT-q) PCR was performed. The cells were cultured under serum-starved conditions at 37 °C overnight and then treated with the indicated concentration of each MTAs or dissolved in dimethyl sulfoxide (DMSO) for 24 h. The extracted RNA from cultured cells using an Isogen kit (Nippon Gene Co., Ltd, Tokyo, Japan) was reversely transcribed into cDNA, and RT-qPCR was performed to detect the mRNA levels of *CD274* (*PD-L1*; programmed cell death ligand 1). Single-strand cDNA was synthesized using a high-capacity RNA-to-cDNA kit (Applied Biosystems; Thermo Fisher Scientific, Inc., Waltham, MA, USA). The thermocycling parameters were as follows: 2 min at 50 °C and 10 min at 95 °C, followed by 45 cycles of 15 s at 95 °C, and 1 min at 60 °C. Relative quantification of gene expression was performed according to the 2^−ΔΔ^CT method using StepOne Software 2.0 (Applied Biosystems). Glyceraldehyde-3-phosphate dehydrogenase (*GAPDH*) was used as a reference for normalization of the target gene expression data. The primers used were purchased from TaqMan^™^ (Applied Biosystems, Foster City, CA, USA) and were as follows: *GAPDH*, Hs02758991_g1; *Gapdh*, Mm99999915_g1; *Cd274*, Mm00452054_m1; *CD274*, Hs00204257_m1.

#### Establishment of the immune syngeneic orthotopic HCC mouse models and animal experiments

Five-week-old female C57BL/6 J mice were purchased from Kyudo KK (Fukuoka, Japan) and housed in cages. Each cage contained six or fewer mice per group at the animal facility of Kurume University School of Medicine (Kurume, Fukuoka, Japan). In total, 2 × 10^6^ Hep-55.1C or Hep-53.4 cells were suspended in 1:1 phosphate-buffered saline (PBS) plus Matrigel matrix (Corning, NY, USA) and orthotopically inoculated into the left lobe of the liver to establish an immune syngeneic orthotopic Hep-55.1C or Hep-53.4 mouse model.

Two weeks after hepatoma cell transplantation, the tumor-bearing mice were allocated to different treatment groups (*n* = 5–6). The treatment groups were as follows: (I) Sorafenib; sorafenib tosylate (CAS No.: 475207-59-1; purchased from Santa Cruz Biotechnology, Inc., TX, USA), administered 30 mg/kg orally/once daily; (II) Lenvatinib; lenvatinib mesylate (CAS No.: 417716-92-8; kindly provided by Eisai co. ltd., Tokyo, Japan), administered 10 mg/kg/mouse orally/once daily; (III) VEGFR2 (VEGF receptor 2) antibody; DC101, an anti-VEGFR2 antibody, purchased from Bio X Cell (Lebanon, NH, USA), administered intraperitoneally thrice/week at a dose of 40 mg/kg; (IV) Regorafenib; regorafenib (CAS No.: 755037-03-7; purchased from Tokyo Chemical Industry, Tokyo, Japan), administered 3 mg/kg orally/once daily; (V) Cabozantinib; cabozantinib malate (CAS No.: 849217-68-1; purchased from ChemScene, Monmouth Junction, NJ, USA), administered 30 mg/kg orally/once daily; (VI) FGFR (FGF receptor) inhibitor; AZD4547, FGFR-1/2/3/4 inhibitor (CAS No.: 1035270-39-3; purchased from ChemScene), administered 12.5 mg/kg intraperitoneally/once daily; (VII) VT (vehicle treatment); PBS as vehicle was administered orally or intraperitoneally. The mice were euthanized by cervical dislocation under anesthesia with isoflurane and pentobarbital two weeks after treatment initiation, and the tumors were resected and evaluated. We calculated the estimated tumor volume as 0.52 × length × width^2^.

#### Immunohistochemical staining

Five-μm-thick of paraffin-embedded tumor tissue sections were boiled for 30 min in a high-pH target retrieval solution for antigen retrieval, followed by incubation with the primary antibodies followed by secondary antibodies. The antibodies used are listed in Supplementary Table S1. To visualize the immunoreactivity, we used an EnVision + system (DAKO North America, Carpinteria, CA, USA) and diaminobenzidine commercial kit (Liquid DAB + Substrate Chromogen System; Dako North America). To quantify CD3-ε-, CD8-α-, Foxp3-, NK1.1-, Granzyme B-, F4/80-, Arginase-1-, CD11c-, programmed cell death-1 (PD-1)-, PD-L1-positive cells, and CD31-positive tumor microvessels were counted at a magnification of × 200 (*n* = 5–10 random fields per group). We defined these positive cells as follows; CD3, pan T cells; CD8, cytotoxic T cells; Foxp3, Tregs; NK1.1, natural killer (NK) cells; Granzyme B, activated cytotoxic T cells or NK cells; F4/80, pan macrophages; Arginase-1, M2 macrophages; CD11c, dendritic cells (DCs). We counted PD-1-positive non-tumor cells and PD-L1-positive tumor cells. All slides were photographed using a confocal microscope BZ-X700 (Keyence Corporation, Osaka, Japan) and quantified using Adobe Photoshop CC 2021 version 22.2.0 (Adobe Systems, Inc., San Jose, CA, USA).

#### Total RNA isolation and RNA sequencing data analysis

From the resected tumor tissues, total ribonucleic acid (RNA) was isolated using TRIzol Reagent (Thermo Fisher Scientific, Inc., MA, USA) and then purified using the SV Total RNA Isolation System (Promega, WI, USA), according to the manufacturer’s instructions. We quantified RNA samples using ND-1000 spectrophotometer (NanoDrop Technologies, Wilmington, DE, USA) and confirmed the quality using TapeStation (Agilent Technologies, Inc., Santa Clara, CA, USA). Using the MGIEasy rRNA Depletion Kit and MGIEasy RNA Directional Library Prep Set (MGI Tech Co., Ltd., Shenzhen, China), sequencing libraries were prepared from 200 ng of RNA. The libraries were sequenced on a DNBSEQ-G400 FAST Sequencer (MGI Tech Co., Ltd.) with a paired-end 150 nt strategy. Quality trimming and adapter clipping of the read data were performed using Trimmomatic version 0.38. Trimmed reads were mapped to the transcript in the reference human hg38 using the Bowtie2 aligner within RNA-Seq by Expectation–Maximization (RSEM). Estimation of gene and isoform abundance using RSEM-generated basic count data (expected count). We used edgeR to detect differentially expressed genes (DEGs). Normalized counts per million (CPM), log-fold change (logFC), and *p *values were obtained from normalized CPM values. Subsequently, the criteria for DEGs were established as follows: *p *value ≤ 0.05, logFC ≥ 1. A heat map of differentially expressed genes was generated using MeV software. Hierarchical clustering was used to sort the genes. The color indicates the distance from the median of each row. The distance metric was “Pearson correlation,” and the linkage method was “average linkage clustering.” Gene functional enrichment was analyzed using the database for annotation, visualization, and integrated discovery [10].

#### Microarray data information

The human RNA sequencing data used in this study were obtained from the Gene Expression Omnibus (GEO) database (http://www.ncbi.nlm.nih.gov/gds/), which is a public repository containing high-throughput gene expression data. We selected GSE214324 from the GEO database, and the array data comprised MHCC-97H cells, HCC cell line, treated with various doses of lenvatinib (40, 60, 80 and 100 μM) or DMSO for 24 h.

### Statistical analyses

All data are expressed as mean ± standard error of the mean (SEM). Differences between two groups were examined for statistical significance using unpaired Student’s *t* test, and differences among multiple groups were examined using one-way analysis of variance (ANOVA), followed by Fisher’s least significant difference test. *p* was set than 0.05. Data analysis was performed using JMP Pro 16.0 software (JMP, Tokyo, Japan).

## Results

### Each MTA decreased tumor volume

First, we examined the antitumor effects of MTAs, which are standard therapies for HCC. Two weeks of administration of these drugs in each treatment group significantly decreased the tumor volume compared to that in the VT group (Fig. S1A, B). As MTAs exert antitumor effects via antiangiogenic effects, we conducted immunohistochemical analyses to evaluate tumor microvessels (CD31-positive cells). A robust reduction in tumor microvessels was observed in all the treatment groups (Fig. S1C, D).

### Lenvatinib, but not sorafenib, alters the immune hot TIME

**Fig. 1:**
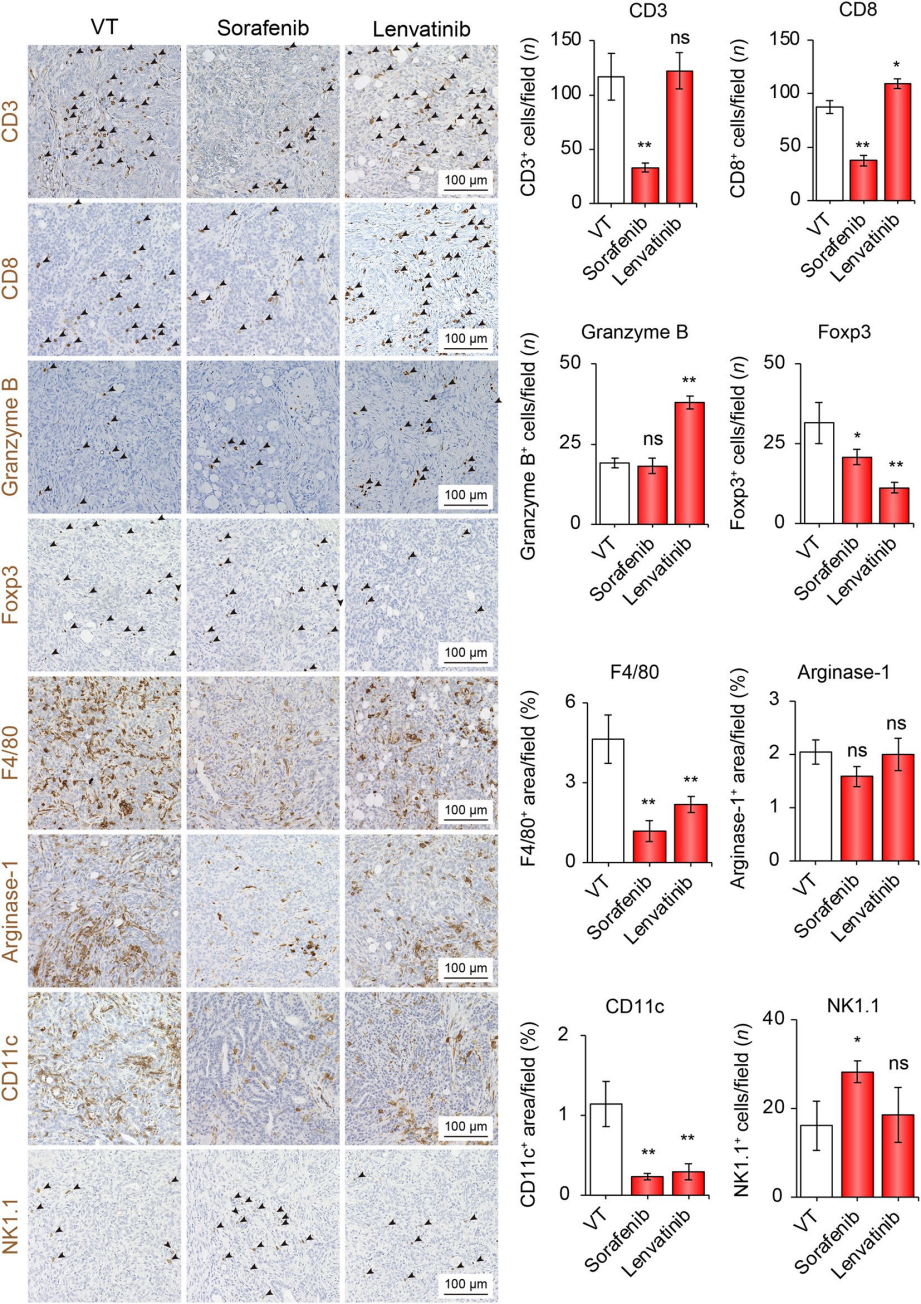
Sorafenib or lenvatinib treatment altered the tumor immune microenvironment. Representative micrographs (left panel) and quantification (right panel) of CD3-positive, CD8-positive, Granzyme B-positive, Foxp3-positive, F4/80-positive areas, Arginase-1-positive areas, CD11c-positive, and NK1.1-positive cells in the VT, sorafenib, and sorafenib groups, respectively. *p < 0.05, **p < 0.01 vs. VT, one-way ANOVA. Data are presented as mean ± SEM. *VT* vehicle treatment, *ns* no significance

Since sorafenib and lenvatinib have been approved as first-line systemic chemotherapies for patients with advanced HCC, we first evaluated the TIME alterations caused by these drugs. In the sorafenib group, compared to the VT group, the numbers of CD3, CD8, Foxp3, F4/80, and CD11c were significantly decreased by 0.28-, 0.42-, 0.30-, 0.21-, and 0.17-time in number/field, respectively. In contrast, NK1.1-positive cells in the sorafenib group significantly increased by 1.75-time in number/field compared to those in the VT group (Fig. 1). In contrast, in the lenvatinib group, positive cells for CD8 and Granzyme B were significantly increased by 1.26- and 1.95-time in number/field compared to those in the VT group, respectively. Foxp3-, F4/80-, and CD11c-positive cells were decreased by 0.27-, 0.39-, and 0.21- time in number/field, respectively. The number of positive cells for Arginase-1 was not changed by sorafenib or lenvatinib treatment. Taken together, these results suggest that lenvatinib might alter the TIME into hot status by increasing the number of infiltrating T cells that attack the tumor cells in this immune syngeneic HCC mouse model using Hep-55.1C cells.

### Cabozantinib altered TIME into hot through the activation of the innate immune system

**Fig. 2:**
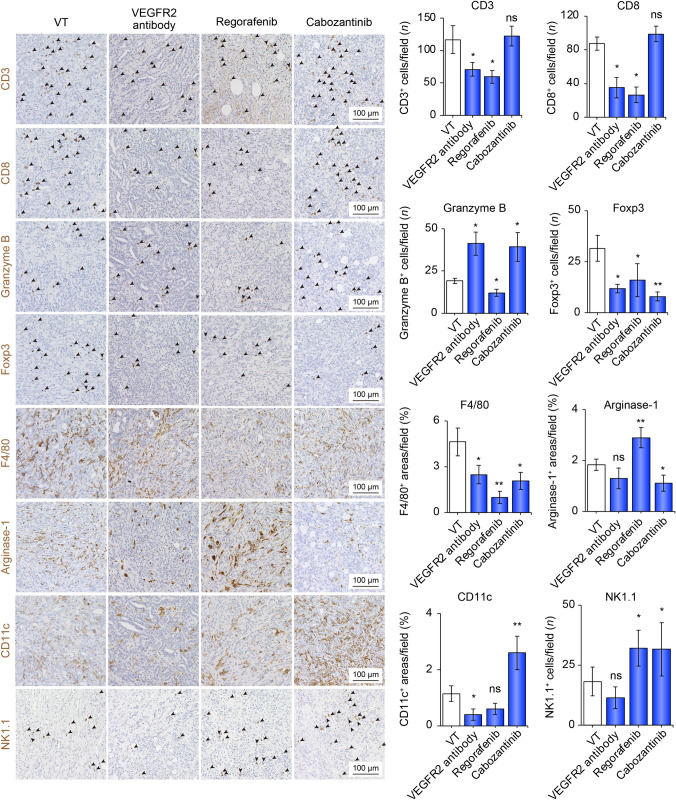
The tumor immune microenvironment was altered by treatment with anti-VEGFR-2 antibody, regorafenib, or cabozantinib. Representative micrographs (left panel) and quantification (right panel) of CD3-positive, CD8-positive, Granzyme B-positive, and Foxp3-positive cells; F4/80-positive, Arginase-1-positive,and CD11c-positive areas; and NK1.1-positive cells in the VT, VEGFR2 antibody, regorafenib, and cabozantinib groups. Arrowheads represent positive cells. *p < 0.05, **p < 0.01 vs. VT, one-way ANOVA. Data are presented as mean ± SEM. *VT* vehicle treatment, *VEGFR2* vascular endothelial growth factor receptor 2, *ns* no significance

Next, we assessed other MTAs, including anti-VEGFR-2 antibody (used in the same manner as ramucirumab in humans), regorafenib, and cabozantinib, which have been approved as second-line drugs for the treatment of advanced HCC. Compared to those in the VT group, the number of cells positive for CD3, CD8, Foxp3, and F4/80 was significantly decreased in the regorafenib and VEGFR2 antibody groups. Compared to the VT group, Granzyme B-positive cells in the cabozantinib and VEGFR2 antibody groups significantly increased by 2.15- and 2.05-time in number/field, respectively (Fig. 2). In the regorafenib and cabozantinib groups, NK1.1-positive cells significantly increased by 2.85- and 1.95-time in number/field, respectively, compared to the VT group. Compared to the VT group, CD11c-positive cells were significantly increased by 2.28 times in number/field only in the cabozantinib group. Thus, cabozantinib can change TIME into the immune system through activation of the innate immune system.

### MTA decreased PD-1 expression, whereas lenvatinib and VEGFR2 antibody treatment increased PD-L1 expression

**Fig. 3:**
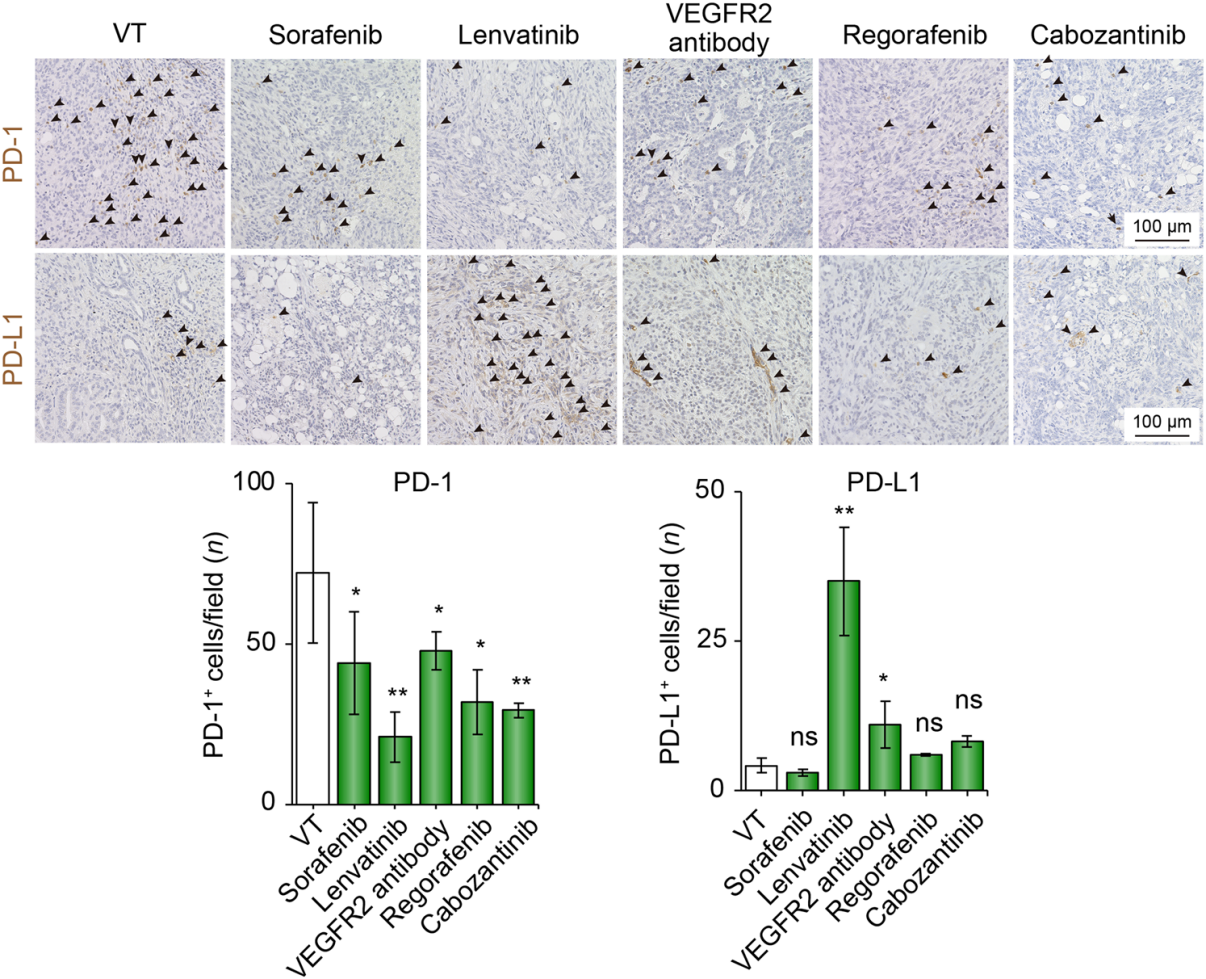
Immune checkpoint molecules altered by MTA treatment. Representative micrographs (upper panel) and quantification (lower panel) of PD-1- and PD-L1-positive cells in VT, sorafenib, lenvatinib, VEGFR2 antibody, and regorafenib cabozantinib groups. Arrowheads represent positive cells. *p < 0.05, **p < 0.01 vs. VT, one-way ANOVA. Data are presented as mean ± SEM. *VT* vehicle treatment, *VEGFR2* vascular endothelial growth factor receptor 2, *ns* not significant, *PD-1* programmed cell death-1, *PD-L1* programmed cell death ligand 1

In addition to assessing the immune cells infiltrating the TIME, we evaluated the expression of immune checkpoint molecules, including PD-1 and PD-L1, in the TIME. Surprisingly, compared with the VT group, PD-1-positive cells, which are known to be markers of exhausted immune cells, were significantly decreased in all treatment groups (Fig. 3). In contrast, PD-L1-positive cells in the lenvatinib and VEGFR2 antibody groups were significantly increased compared with those in the VT group (Fig. 3). We analyzed the impact of MTAs on PD-L1 expression in human (HuH7) and mouse (Hep-55.1C) hepatoma cell lines and found no significant differences among these MTAs (Fig. S2).

### The FGFR-1/2/3/4 inhibitor altered the TIME into the immune hot

**Fig. 4:**
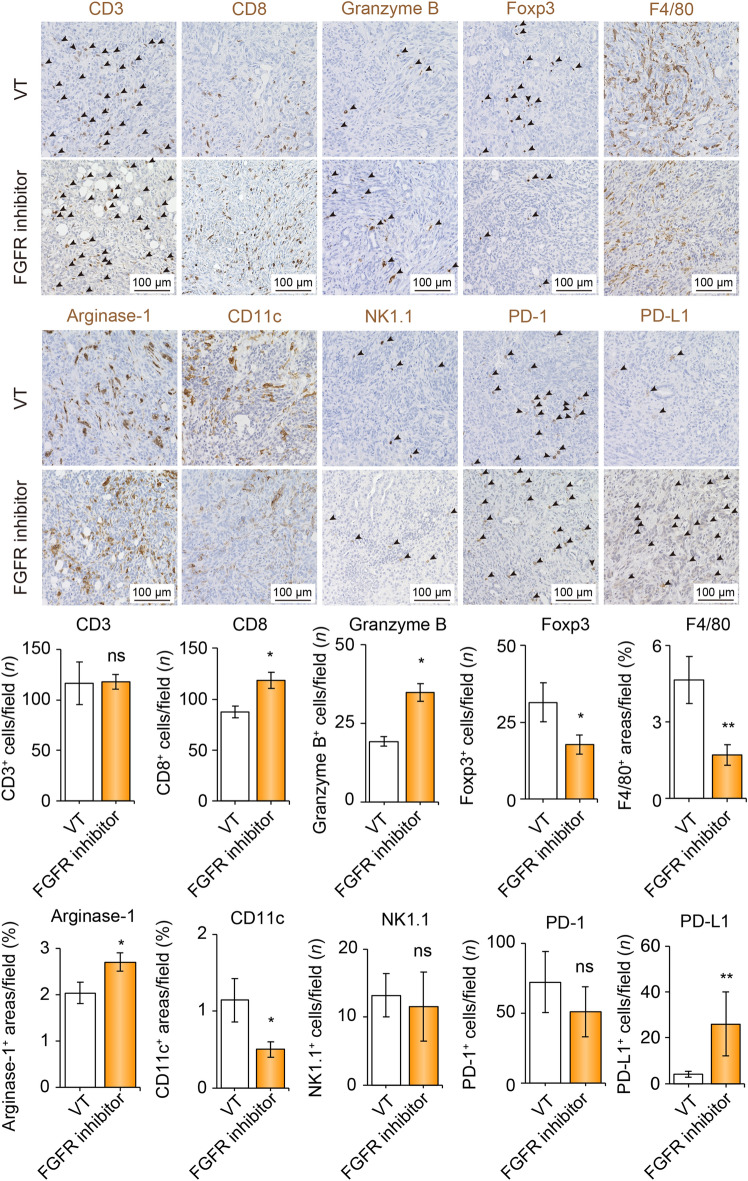
Tumor immune microenvironment is altered by FGFR-1/2/3/4 inhibitor treatment. Representative micrographs (upper panel) and quantification (lower panel) of CD3-positive, CD8-positive, Granzyme B-positive, Foxp3-positive, F4/80-positive areas, Arginase-1-positive areas, CD11c-positive, NK1.1-positive, PD-1-, and PD-L1-positive cells in the VT and FGFR inhibitor groups, respectively. Arrowheads represent positive cells. *p < 0.05, **p < 0.01 vs. VT, Student *t *test. Data are presented as mean ± SEM. *VT* vehicle treatment, *FGFR* fibroblast growth factor receptor, *ns* not significant, *PD-1* programmed cell death-1, *PD-L1* programmed cell death ligand 1

As lenvatinib treatment increased CD8- and granzyme B-positive cells and concomitantly decreased Foxp3- and PD-1-positive cells in the TIME, it was considered to alter the TIME to an immune hot state. Compared with other MTAs, lenvatinib has characteristically selective and strong FGFR-inhibitory effects. Therefore, we tested the Hep-55.1C orthotopic model treated with AZD4547, an FGFR-1/2/3/4 inhibitor (Fig. 4). In the FGFR inhibitor group, compared to the VT group, positive cells for CD8 and Granzyme B were significantly increased by 1.38- and 1.81- times in number/field, respectively. In addition, Foxp3-, F4/80-, and CD11c-positive cells decreased by 0.42-, 0.29-, and 0.35-time in number/field, respectively. Taken together, FGFR signaling blockade may alter the TIME in an immune hot state. To validate the effects of lenvatinib and an FGFR inhibitor, we generated another mouse model using the cell line, Hep-53.4 (Fig. S3). Compared with the findings from Hep-55.1C model, there were no significant differences in none of immune cells indicated by these surface markers, whereas, the CD3-, CD8-, and F4/80-positive cells were relatively low levels in the Hep-53.4 group. Notably, a similar alternation, where upregulation of CD8- and Granzyme B-positive cells with decreased Foxp3-positive cells was observed in these experiments.

### Lenvatinib or FGFR-1/2/3/4 inhibitor treatment commonly downregulated metabolic pathway signaling

**Fig. 5:**
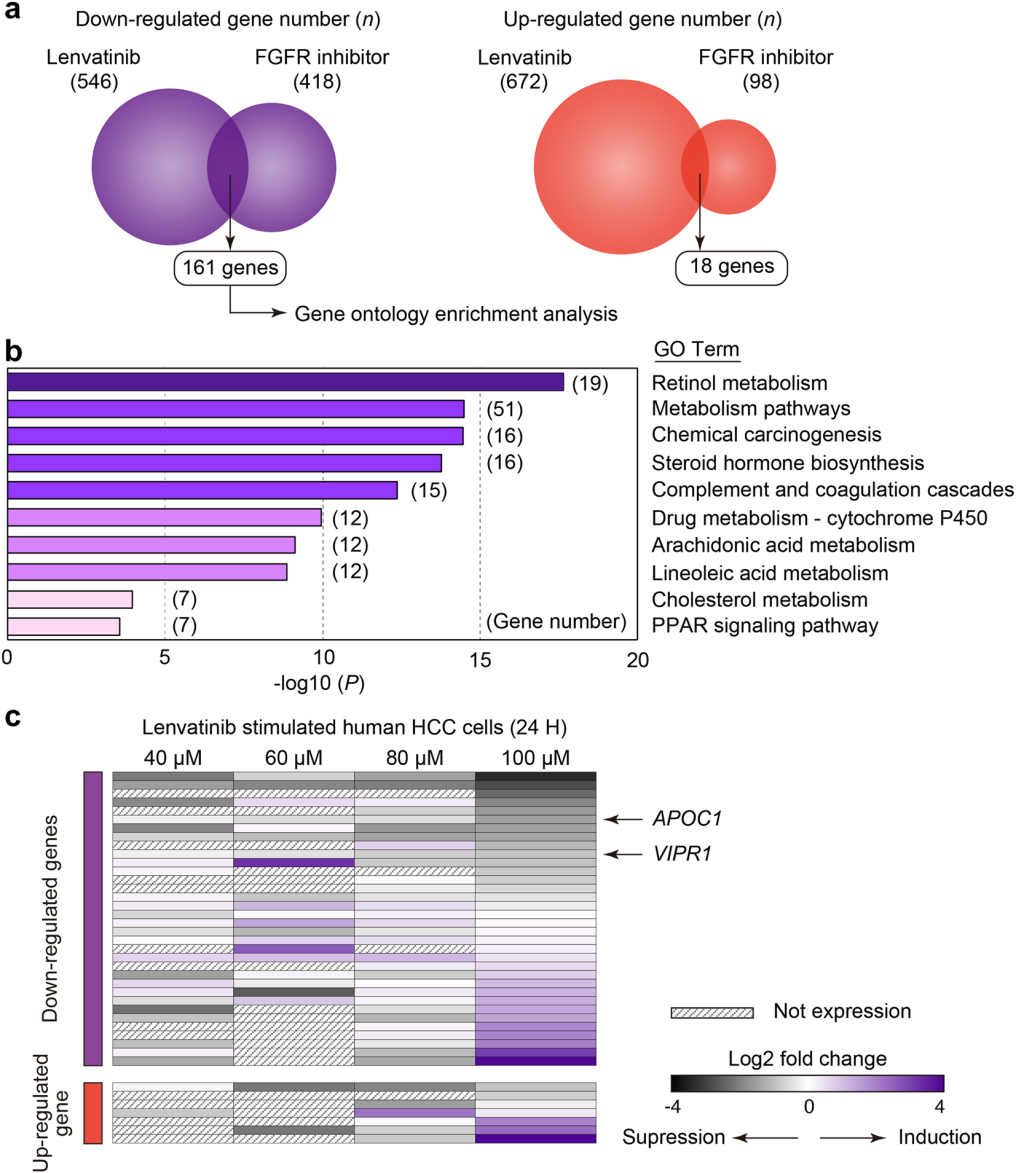
Genes commonly altered between lenvatinib and FGFR inhibitor groups and lenvatinib-induced gene alterations in human HCC. **a** Venn figure for common up- and downregulated gene numbers between the lenvatinib and FGFR inhibitor groups (*n* = 3/group). **b** Gene ontology enrichment analysis of 161 commonly downregulated genes in the lenvatinib and FGFR inhibitor groups. **C** Restructured human RNA sequencing data using GSE214324 from the GEO database. Gene expression in MHCC-97H cells treated with various doses of lenvatinib (40, 60, 80, and 100 μM) compared to that in dimethyl sulfoxide-stimulated cells. *FGFR* fibroblast growth factor receptor, *VT* vehicle treatment, *VEGFR* vascular endothelial growth factor receptor, *PPAR* peroxisome proliferator-activated receptor

To reveal the underlying signals implicating the alteration of TIME by FGFR inhibition, we conducted RNA-seq analysis of lenvatinib- and FGFR inhibitor-treated HCC. To explore the commonly altered genes involved in the immune system between these groups, we generated heat maps of the signaling pathways for ‘T cell receptor’, “NK cell-mediated cytotoxicity,” ‘Th1/Th2 differentiation’, “antigen processing and presentation,” ‘PD-L1 expression and PD-1 checkpoint,’ and ‘Wnt’ (Fig. S4). However, genes that concentrated on specific signals did not appear in these pathways. The number of upregulated and downregulated genes in the lenvatinib and FGFR inhibitor groups was 546/672 and 418/98, respectively. Between the lenvatinib and FGFR inhibitor groups, 161 and 18 genes were downregulated and upregulated, respectively (Fig. 5A). Using 161 commonly downregulated genes, we conducted gene ontology (GO) enrichment analysis. GO enrichment analysis revealed that GO terms such as ‘retinol metabolism,’ ‘metabolic pathways,’ ‘steroid hormone biosynthesis,’ and ‘PPAR signaling pathway’ were enriched (Fig. 5B).

### Inhibition of FGFR signaling with lenvatinib leads to suppression of metabolic pathways in human HCC cells

To confirm the clinical relevance of these findings, we analyzed the transcriptome data of human HCC cells (MHCC-97H) treated with lenvatinib for 24 h at various concentrations. Notably, 161 genes downregulated by FGFR inhibition in rodent models overlapped with those downregulated by lenvatinib in human HCC cells (Fig. 5C). *APOC1* and *VIPR1* are among the genes that exhibited dose-dependent suppression. Genetic knockout of *Apoc1* leads to ferroptosis-mediated M2 to M1 macrophage polarization and enhances the efficacy of anti-PD1, accompanied by increased CD8-positive T cells in a mouse HCC model [11]. *VIPR1* induces naïve CD4-positive T cells to express FoxP3-generating inducible Tregs and contributes to the development of an immunosuppressive tissue microenvironment [12, 13].

## Discussion

**Fig. 6:**
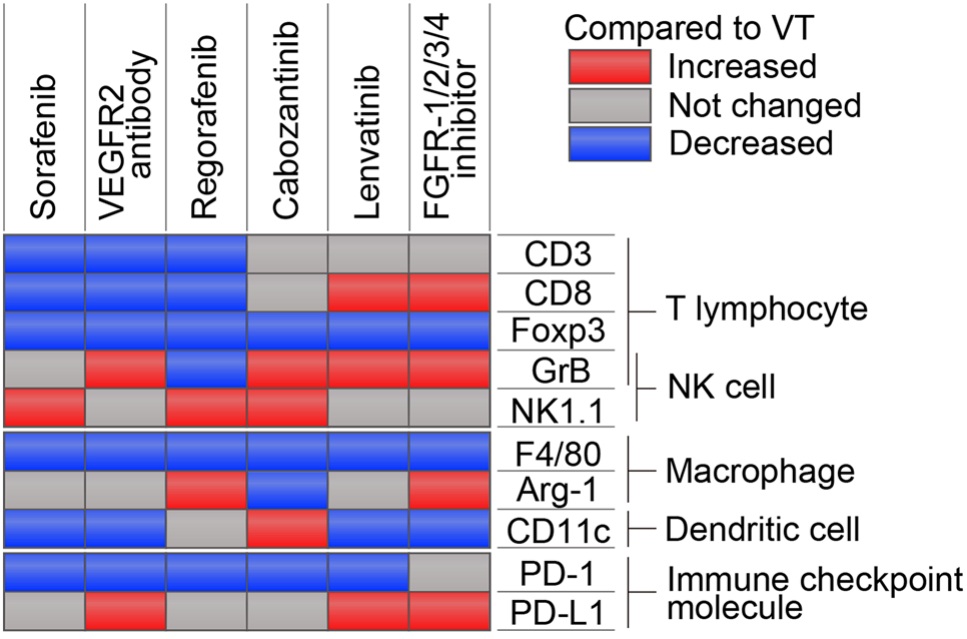
Overview of this study. The summarized changes of TIME caused by MTAs in immune syngeneic orthotopic Hep-55.1C mouse model. Compared to the VT group, significantly upregulated/downregulated each cells in each treatment group were shown in red/blue. *VT* vehicle treatment, *VEGFR* vascular endothelial growth factor receptor, *FGFR* fibroblast growth factor receptor, *NK* natural killer, *PD-L1* programmed cell death ligand 1, *PD-1* programmed cell death-1

In this study, we uncovered distinct effects of MTAs on the TIME in immune syngeneic orthotopic HCC mouse models. In many MTAs, the number of Tregs and macrophages in the TIME decreased. Cabozantinib, characterized by inhibition of c-MET and AXL, might promote the innate immune systems via activation and infiltrating of NK cells and DCs. Inhibition of FGFR signaling by lenvatinib and FGFR-1/2/3/4 inhibitor altered the TIME into immune hot (Fig. 6).

Consistent with our study, previous studies which evaluated the immunomodulatory effects of lenvatinib in real-world clinical practice in patients with HCC have shown that lenvatinib might alter the TIME to immune hot. Analysis of blood samples showed an increase in cytotoxic T cells and a decrease in Tregs one month after lenvatinib treatment [14]. In addition, a report using surgical specimens successfully converted after lenvatinib treatment showed that the number of CD8-positive T cells (especially Granzyme K/CD8-positive T cells) was significantly higher in tumor samples after lenvatinib treatment than in the control [15]. This study reported no significant differences in Tregs. However, there have been no reports on increases/decreases in the number of macrophages and/or PD-1-positive cells using clinical specimens.

Although great advances and paradigm changes have been made in HCC treatment, the antitumor effects of ICI alone/combination immunotherapy remain limited (up to 30%) [2]. Tumor control is difficult with these therapies alone; therefore, in real-world practice, sequential therapy using various MTAs and ICIs, without impairing liver reserves, aims to prolong PFS and OS [8, 16]. A recent retrospective study regarding outcomes of subsequent therapy after ICI discontinuation, it was shown that receipt of post-ICI therapy including MTAs was associated with longer median OS compared with those who had received the best supportive care (12.1 vs. 3.3 months; hazard ratio: 0.4; 95% confidence index: 2.7–5.0) [17]. Another study shows that in patients with advanced HCC after progressive disease on atezolizumab plus bevacizumab, the median PFS of second-line treatment with lenvatinib was significantly longer than that of sorafenib (6.1 vs. 2.5 months; *p* = 0.004) [18]. Several studies in real-world clinical practice have shown that ICI (atezolizumab along with bevacizumab) after lenvatinib treatment is less effective than ICI before lenvatinib (objective response rate: 12.2–17.4% vs. 7.7–12.5%) [19–21]. It is presumed that the patient background, such as liver function, is better when initiating ICI as primary therapy compared to introducing ICI as secondary therapy. Thus, not only lenvatinib, but any MTAs in the previous treatment would be less effective when ICI is used in the secondary or later treatment. In this study, although we evaluated the status of TIME at the time of antitumor efficacy exerted by MTA including lenvatinib, it is unclear whether immune activation is maintained at the time of PD after lenvatinib treatment. In addition, the biological inhibitory effects of lenvatinib would disappear after 2 days of no treatment [22], therefore, it was suggested that the effect of lenvatinib on TIME might have decayed by the time of ICI induction. Appropriate sequential therapy following ICIs therapy remains controversial, and a better understanding of the alteration of TIME by these drugs in sequencing therapy is of utmost importance.

VEGF is a protein that allows tumor cells to develop blood vessels to facilitate the supply of nutrients and oxygen. Although inhibition of VEGF signaling is thought to have the primary effect of inhibiting angiogenesis, it has recently been shown that VEGF also promotes tumor cell metastasis, epithelial–mesenchymal transition, and alteration of the TIME favorable to tumor cells [23]. VEGF plays a significant role in TIME formation, including macrophage recruitment, M2 polarization, Treg recruitment, myeloid-derived suppressor cell recruitment and activation, and inhibition of DC maturation [24]. As the MTAs used in the current study commonly have inhibitory effects on VEGFR signaling, these treatments might reduce the number of macrophages and Tregs.

FGFs, encoded by 22 genes, bind to and activate alternatively spliced forms of four tyrosine kinases, FGFR-1/2/3/4. FGFs are known to be involved in TIME formation (for example, FGF2 alters macrophage polarization) [25]. A recent study revealed that targeting DNA methyltransferase 1 (DNMT1) programs immunologically anergic tumor vasculature, and its phosphorylation is promoted by FGF2 [26]. Several studies on FGFs have reported immunomodulatory effects by impacting various stages of the “cancer-immune cycle”; therefore, FGFR inhibitors are believed to be among the most promising MTAs paired with ICIs [27–32]. Consistent with previous studies showing that FGFR signaling inhibition results in increased cytotoxic T cell infiltration and decreased regulatory Tregs/macrophages, our study also showed these phenomena [27–32]. However, particularly regarding the expression of PD-L1, these studies have reported both elevated and decreased expression; thus, it is controversial. In the present study, we uncovered that MTAs did not directly upregulate the PD-L1 expression in HCC cells (Fig. S2). PD-L1 expression in TIME is known to be regulated by various factors at the levels such as transcription, post-transcription, post-translation. Therefore, the underlying mechanism of PD-L1 elevation in TIME caused by MTAs was thought to be derived from multiple factors including cell–cell interactions, hypoxia in tumor microenvironment [33]. GO analysis was used to elucidate the underlying mechanism of the FGFR signaling blockade, and the main mechanism was found to be the inhibition of metabolic pathway signaling. Zhewen et al. showed that higher baseline PPARγ expression was associated with poorer survival of ICI-treated patients in multiple cancer types [34]. The depletion of energy (e.g., lipid and glucose) in TIME is known to promote the formation of an immune cold environment, especially affecting T cell function such as exhausting [35–37]. It was suggested that the suppression of lipid metabolic pathways by FGF signal inhibition revealed in this study led the TIME to an immune hot state through the releasing of energy depletion in TIME.

In this study, we established an orthotopic HCC model using two cell lines, Hep-55.1C and Hep-53.4, to validate our results. The number of CD3-positive cells increased in Hep-53.4, but remained unchanged in Hep-55.1C cells. Inconsistent with a previous report that lenvatinib enhances tumor infiltration and NK cell activation, our study did not show an increase in NK cells. Zhang et al. generated a xenograft mouse model using melanoma or renal cell carcinoma cell lines; therefore, differences in the cell lines may result in diversity [38]. Taken together, it is important to consider the differences in the efficacy of MTAs on TIME due to the differences in cell lines.

In the present study, only cabozantinib treatment, which is characterized by the inhibitory effects of c-MET and AXL, increased the number of DCs in the TIME. The AXL receptor is upregulated when DCs are cultured with type I interferons, and its upregulated expression inhibits the inflammatory response of the innate immune system [39]. c-Met expression in the immune system is limited to cells with antigen-presenting capacity, including macrophages and DCs [40]. Therefore, inhibition of AXL signaling is believed to activate the innate immune system. Consistent with a previous study reporting that cabozantinib may exert an immunostimulatory effect by directly modulating DCs, our study revealed that cabozantinib increased the number of DCs [40].

The current study had several limitations. To assess tumor-infiltrating lymphocytes to reduce sampling errors, flow cytometric analysis should be conducted; however, this could not be confirmed because of low cell viability during single-cell suspension preparation. To perform a cross-sectional drug evaluation, we used a single point of assessment 2 weeks after hepatoma cell transplantation. However, multipoint evaluations are required to determine the true effectiveness of these MTAs. As we could evaluate TIME at the point when all drugs exert an antitumor effect, the present study evaluated the impact of MTAs on TIME.

## Conclusion

In orthotopic mouse models of HCC, lenvatinib and FGFR-1/2/3/4 inhibitors, which commonly inhibit FGFR signaling, alter the TIME to an immune hot state by downregulating metabolic pathway signaling. Our findings support the therapeutic concept of combination immunotherapy including MTAs and ICIs.

### Supplementary Information

Below is the link to the electronic supplementary material.

Supplementary file1 (DOCX 15726 KB)

## Data Availability

The data that support the findings of the current study are available from the corresponding author upon reasonable request.

## Abbreviations

HCC: Hepatocellular carcinoma
ICI: Immune checkpoint inhibitor
MTA: Molecular targeted agent
TIME: Tumor immune microenvironment
PFS: Progression-free survival
OS: Overall survival
VEGF: Vascular endothelial growth factor
Treg: Regulatory T cell
FGF: Fibroblast growth factor
DMSO: Dimethyl sulfoxide
GAPDH: Glyceraldehyde-3-phosphate dehydrogenase
DMEM: Dulbecco’s modified Eagle’s medium
PBS: Phosphate-buffered saline
VEGFR2: VEGF receptor 2
VT: Vehicle treatment
PD-1: Programmed cell death-1
PD-L1: Programmed cell death ligand 1
NK: Natural killer
DC: Dendritic cell
RNA: Ribonucleic acid
RSEM: RNA-Seq by expectation–maximization
DEG: Differentially expressed gene
CPM: Counts per million
logFC: Log-fold change
GEO: Gene expression omnibus
SEM: Standard error of the mean
ANOVA: Analysis of variance
GO: Gene ontology
DNMT1: DNA methyltransferase 1
