# Supplementary material for: Fibroblast growth factor inhibition by molecular-targeted agents mitigates immunosuppressive tissue microenvironment in hepatocellular carcinoma
Source: Hepatol Int. 2023 Oct 21;18(2):610–22. doi: 10.1007/s12072-023-10603-z (PMC11014819; doi:10.1007/s12072-023-10603-z)
Supplement: Supplementary file 1 — Supplementary file1 (DOCX 15726 KB) [file 12072_2023_10603_MOESM1_ESM.docx]

***Research Article***

***Fibroblast growth factor inhibition by molecular-targeted agents mitigates immuno-suppressive tissue microenvironment in hepatocellular carcinoma***

Hiroyuki Suzuki^a,b^*, Hideki Iwamoto^a,b,c^*, Toshimitsu Tanaka^a,b^, Takahiko Sakaue^a,b^, Yasuko Imamura^b^, Atsutaka Masuda^a,b^, Toru Nakamura^a,b^, Hironori Koga^a,b^, Yujin Hoshida^d^, and Takumi Kawaguchi^a^

^a^Division of Gastroenterology, Department of Medicine, Kurume University School of Medicine, Kurume, 830-0011, Japan.

^b^Liver Cancer Research Division, Research Center for Innovative Cancer Therapy, Kurume University, Kurume, 830-0011, Japan.

^c^Iwamoto Internal Medicine Clinic, Kitakyushu, 802-0832, Japan.

^d^Division of Digestive and Liver Diseases, Department of Internal Medicine, University of Texas Southwestern Medical Center, Dallas, TX 75390, USA.

Corresponding Authors:

Hiroyuki Suzuki, M.D., Ph.D.

Division of Gastroenterology, Department of Medicine, Kurume University School of Medicine, 67 Asahi-machi, Kurume, 830-0011, Japan.

Phone: +81-942-35-3311, FAX: +81-942-31-7747, E-mail: suzuki_hiroyuki@med.kurume-u.ac.jp

ORCID: https://orcid.org/0000-0003-2383-5038

Hideki Iwamoto, M.D., Ph.D.

Division of Gastroenterology, Department of Medicine, Kurume University School of Medicine, 67 Asahi-machi, Kurume, 830-0011, Japan.

Phone: +81-942-35-3311, FAX: +81-942-31-7747, E-mail: iwamoto_hideki@med.kurume-u.ac.jp

ORCID: https://orcid.org/0000-0001-5688-1335

**Supplementary**

**Supplementary Figure. S1.** Tumor volume measurements and microvessels after MTA treatment.


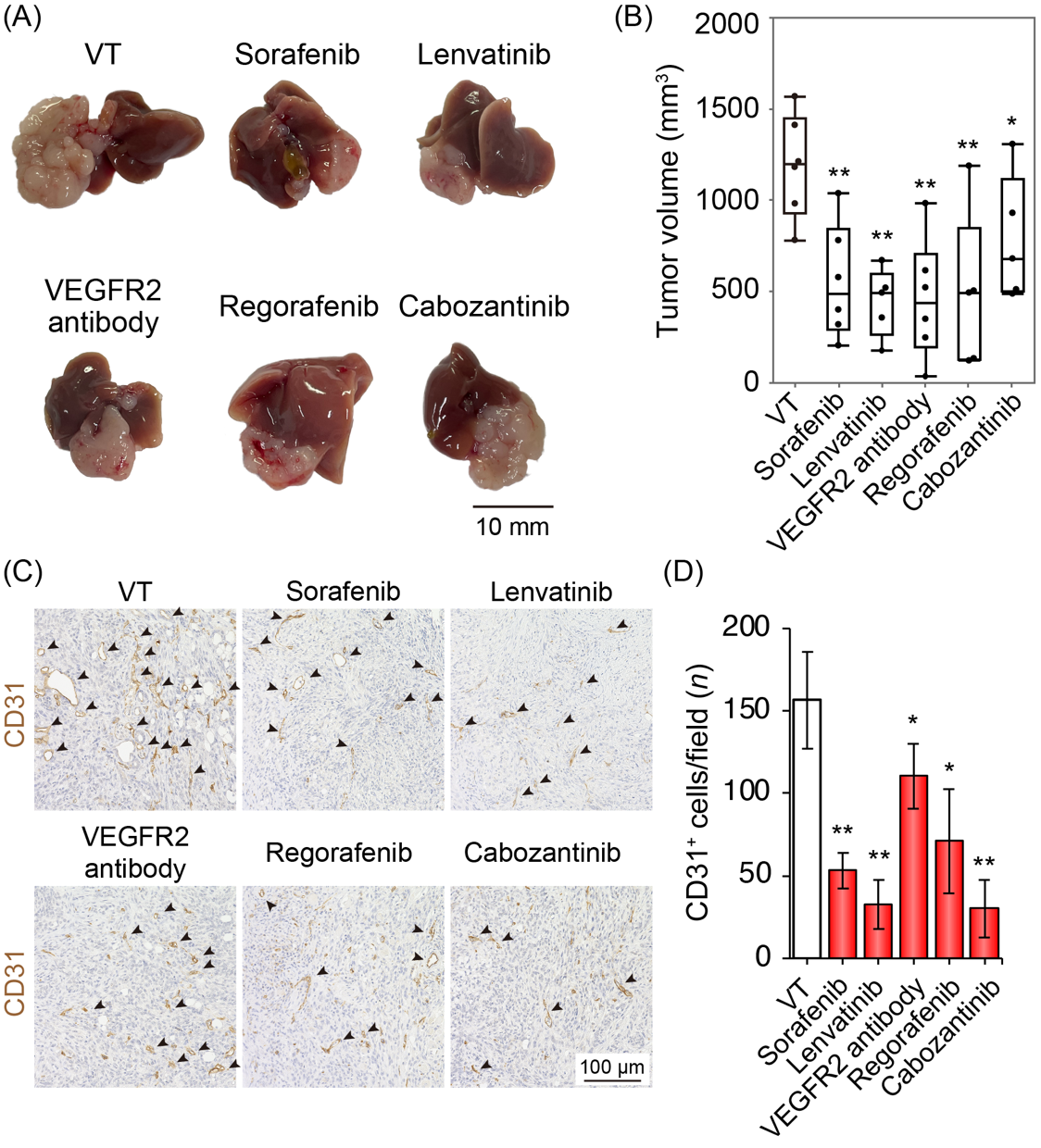


(**A**) Representative macrographs of tumors in each treatment group. (**B**) Quantification of the estimated tumor volume in each treatment group (*n* = 5–6 mice per group). (**C**) Representative micrographs of CD31-positive tumor microvessels. Arrowheads represent CD31-positive tumor microvessels. (**D**) Quantification of CD31-positive tumor microvessels (*n* = 5–10 random fields per group). **P* <0.05, ***P* <0.01 vs. VT, one-way ANOVA. Data are presented as the mean ± SEM.

**Abbreviations:** VT, vehicle treatment; VEGFR2, vascular endothelial growth factor receptor 2.

**Supplementary Figure. S2.** PD-L1 expression in hepatoma cell lines stimulated by MTAs

**
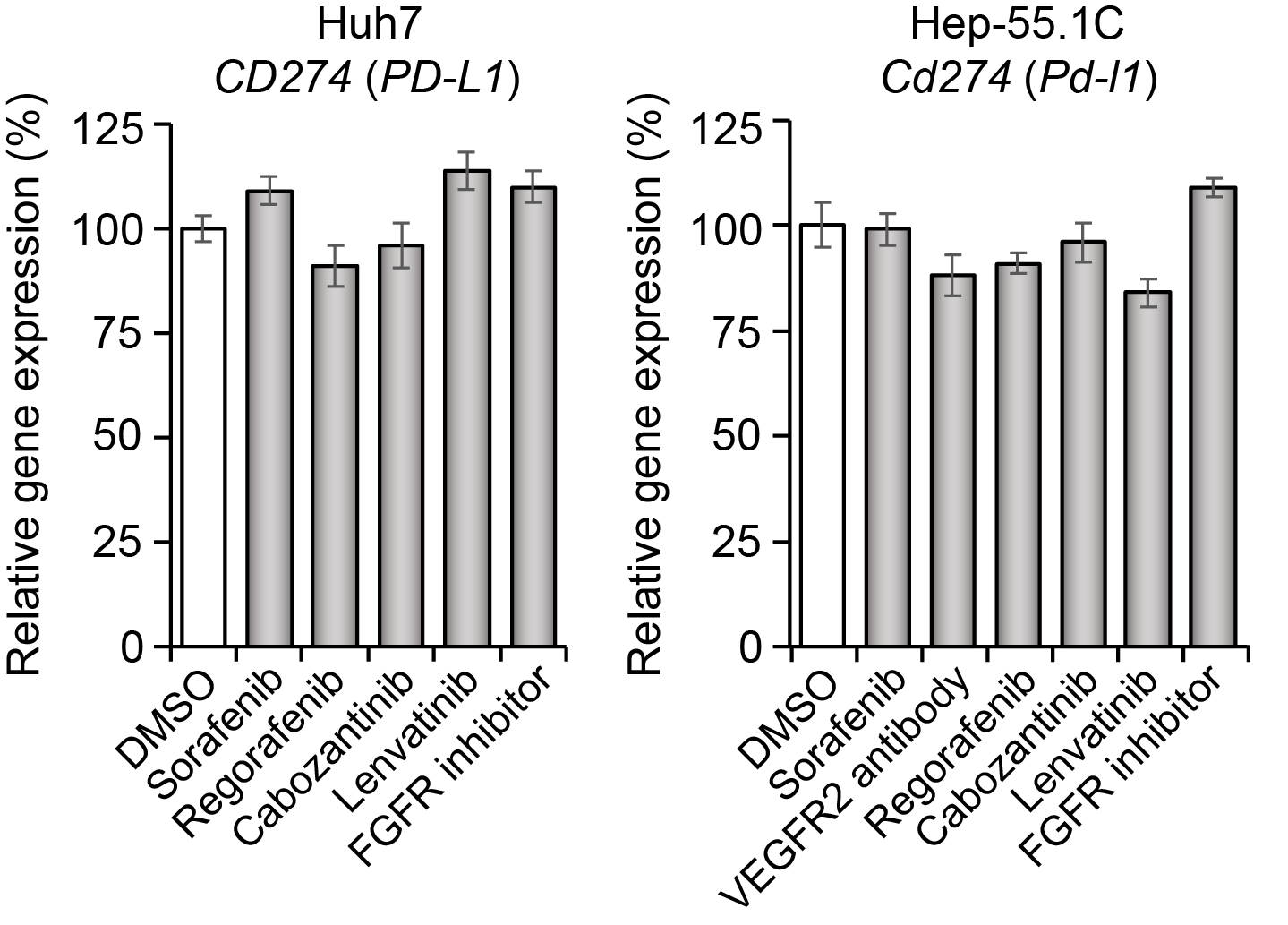
**

Relative gene expression of *CD274* in HuH7 (left panel) and *Cd274* in Hep-55.1C (right panel) stimulated by 24 h of MTAs (sorafenib, 3 μmol/L; regorafenib, 1 μmol/L; cabozantinib, 1 μmol/L; lenvatinib, 3 μmol/L, FGFR inhibitor, 1 μmol/L; VEGFR2 antibody, 3 μmol/L: *n* = 3 per group). There were no significant diffenrences in expression of PD-L1 among the groups (one-way ANOVA). Data are presented as the mean ± SEM.

**Abbreviations:** DMSO, dimethyl sulfoxide; PD-L1, programmed cell death ligand 1; FGFR, fibroblast growth factor receptor; VEGFR2, vascular endothelial growth factor receptor 2.

**Supplementary Figure. S3.** Tumor volume measurements and tumor microvessels after lenvatinib and FGFR inhibitor treatment in the Hep-53.4 orthotopic mouse model**
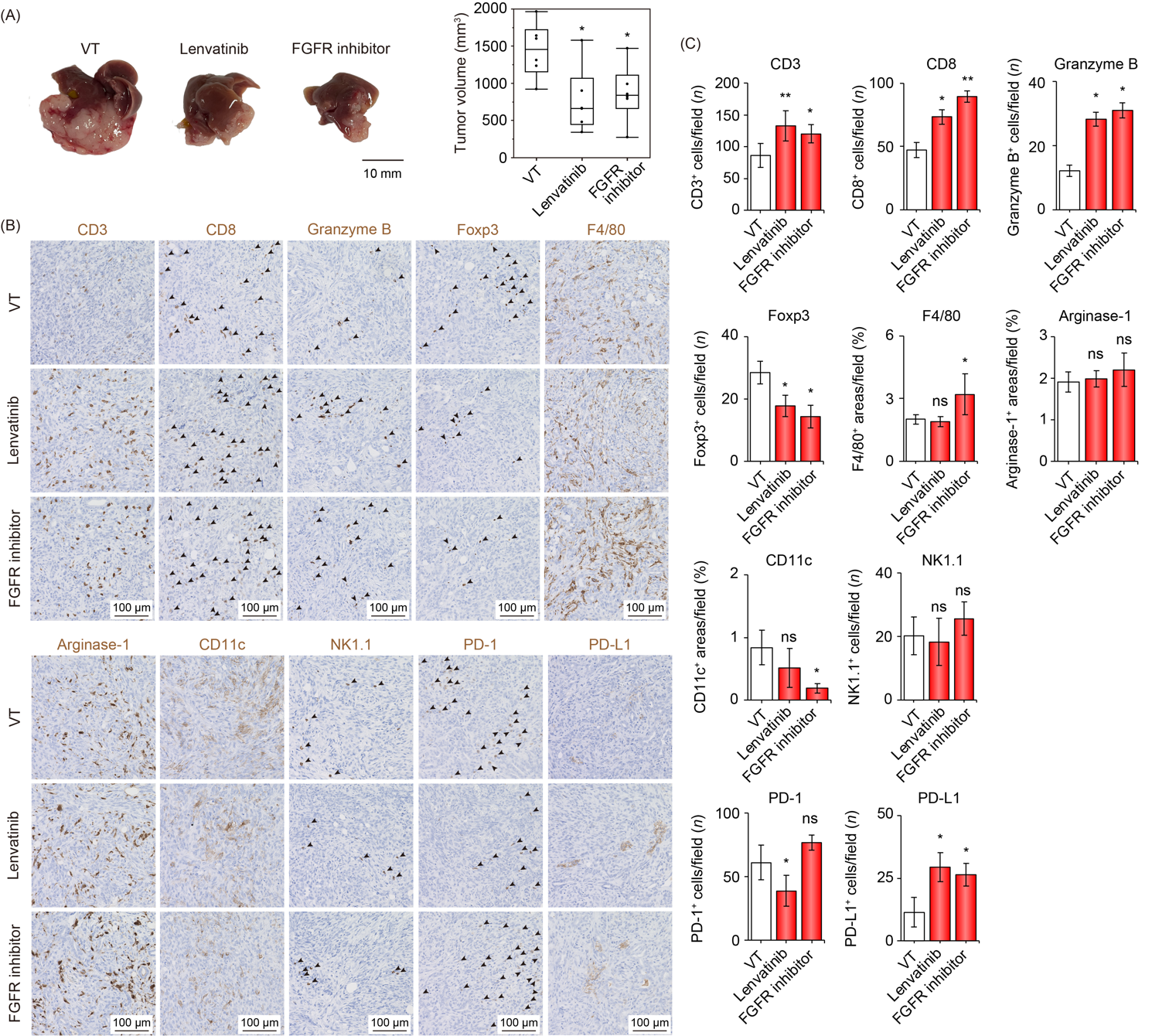
**

(**A**) Representative tumor macrographs (left panel) and quantification of tumor volume in each treatment group (*n* = 5–6 mice/group). (**B-C**) Representative micrographs (**B**) and quantification (**C**) of CD3-positive, CD8-positive, Granzyme B-positive, Foxp3-positive, F4/80-positive areas, Arginase-1-positive areas, CD11c-positive, NK1.1-positive, PD-1-, and PD-L1-positive cells in the VT, lenvatinib, and FGFR inhibitor groups, respectively. Arrowheads represent each-positive cells. **P* <0.05, ***P* <0.01 vs. VT, one-way ANOVA. Data are presented as the mean ± SEM.

**Abbreviations:** VT, vehicle treatment; FGFR, fibroblast growth factor receptor; PD-1 programmed cell death-1; PD-L1, programmed cell death ligand 1, PD-L1; ns, not significant.

**Supplementary Figure. S4.** Heatmaps for signaling pathway analysis between the VT, lenvatinib, and FGFR inhibitor groups in the Hep-55.1C orthotopic mouse model

**
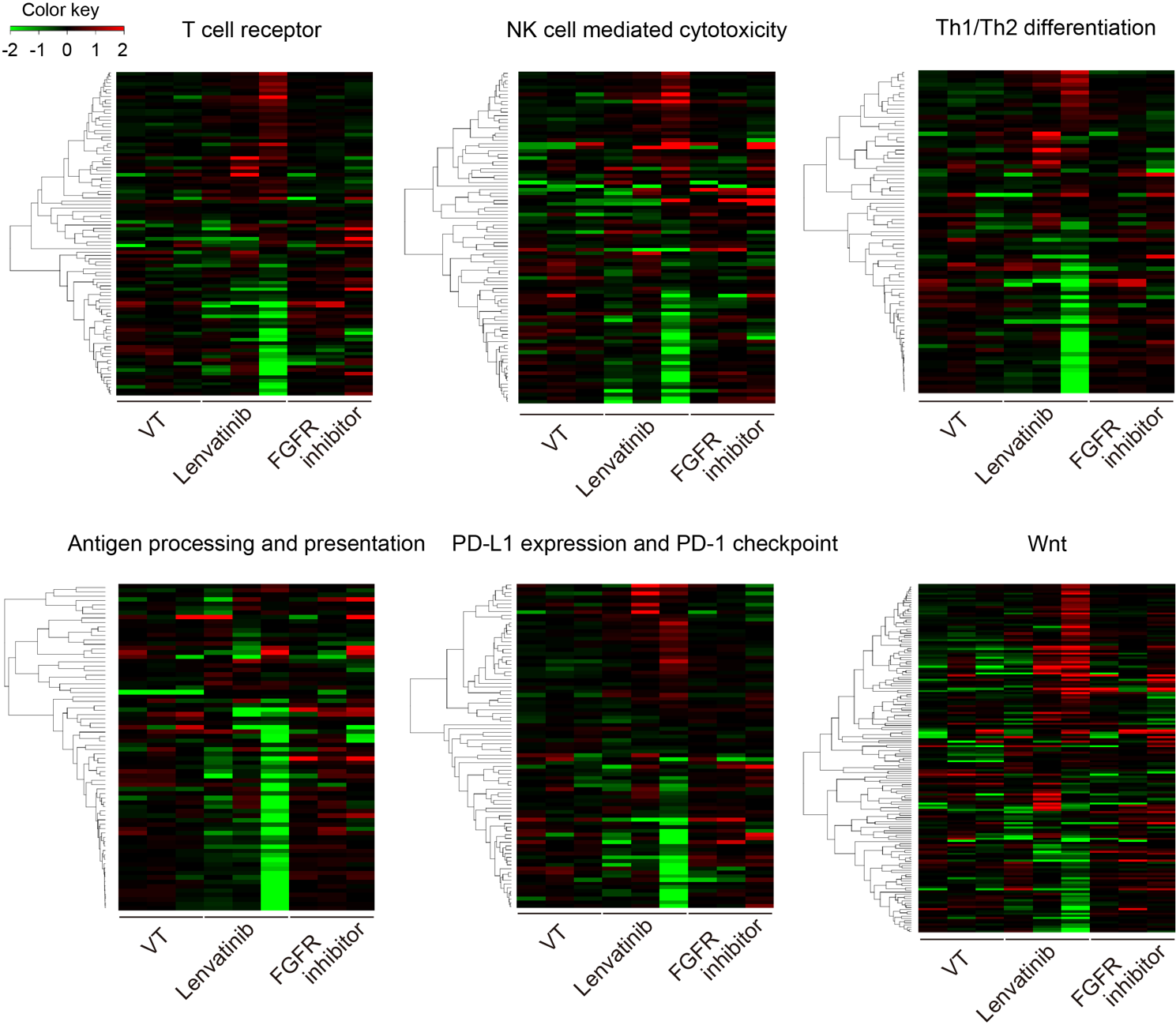
**

**Abbreviations:** VT, vehicle treatment; FGFR, fibroblast growth factor receptor; NK, natural killer**;** Th, helper T; PD-L1, programmed cell death ligand 1; PD-1, programmed cell death-1.

**Supplementary Table S1.** List of antibodies used for immunohistochemistry.

| **Antibody** | **Source** | **Dilution** |
| --- | --- | --- |
| Arginase-1 (D4E3M; rabbit mAb) | #93668; Cell Signaling Technology | 1:400 |
| CD11c (D1V9Y; rabbit mAb) | #97585; Cell Signaling Technology | 1:350 |
| CD3-ε (D4V4B; rabbit mAb) | #99940; Cell Signaling Technology | 1:150 |
| CD31 (Goat pAb) | #AF3628; R&D Systems (Minneapolis, MN, USA) | 1:200 |
| CD8-α (D4W2Z; rabbit mAb) | #98941; Cell Signaling Technology | 1:400 |
| FoxP3 (D608R; rabbit mAb) | #12653; Cell Signaling Technology (Danvers, MA, USA) | 1:250 |
| F4/80 (D2S9R; rabbit pAb) | #70076; Cell Signaling Technology | 1:250 |
| Granzyme B (D6E9W; rabbit mAb) | #46890; Cell Signaling Technology | 1:125 |
| NK1.1 (E6Y9G; rabbit mAb) | #39197; Cell Signaling Technology | 1:200 |
| PD-1 (D7D5W; rabbit mAb) | #84651; Cell Signaling Technology | 1:200 |
| PD-L1 (D5V3B; rabbit mAb) | #64988; Cell Signaling Technology | 1:200 |

Abbreviations: pAb, polyclonal antibody; mAb, monoclonal antibody.
